# Editorial: Beyond protein degradation and lysine modification: novel insights into non-canonical ubiquitination

**DOI:** 10.3389/fmolb.2024.1461356

**Published:** 2024-07-30

**Authors:** Aysegul Sapmaz, Jin Gan, Darragh P. O’Brien, Adán Pinto-Fernández

**Affiliations:** ^1^ Chemical Biology and Drug Discovery Laboratory, Department of Cell and Chemical Biology, Leiden University Medical Center, Leiden, Netherlands; ^2^ Program in Cellular and Molecular Medicine, Boston Children’s Hospital and Harvard Medical School, Boston, MA, United States; ^3^ Structural and Mechanistic Proteomics Laboratory, Nuffield Department of Medicine, Centre for Medicines Discovery, University of Oxford, Oxford, United Kingdom; ^4^ Translational Ubiquitomics Laboratory, Nuffield Department of Medicine, CAMS Oxford Institute and Centre for Medicines Discovery, University of Oxford, Oxford, United Kingdom

**Keywords:** ubiquitin, ubiquitin-like modifiers, activity-based probes, non-canonical ubiquitination, ubiquitomics, chemical biology, post-translational modifications

Protein ubiquitination is a process of post-translational modification, whereby a 76-residue ubiquitin (Ub) protein is covalently attached to lysine residues on target proteins, and is tightly controlled by conjugating (E1, E2, E3) and deubiquitylating (DUBs) enzymes. Several ubiquitin-like (UBL) modifiers including NEDD8, SUMO, ISG15, UFM1, FAT10, and URM1 have also been identified. Since the discovery of the UPS, significant progress has been made in identifying the individual proteins and pathways involved in canonical Ub and UBL protein modification. This has led to a deeper understanding of how cells maintain homeostasis and respond to stress, with important implications for the development of treatments for diseases such as cancer and neurodegeneration.

There is increasing evidence for non-canonical ubiquitination, which deviates from the conventional ubiquitin-proteasome system (UPS) pathway primarily involving lysine-mediated ubiquitination. This includes modifications by UBLs, the attachment of Ub to serine, threonine, or cysteine residues, the modification of non-protein targets, and post-translational modifications of the ubiquitin protein itself. Such non-canonical ubiquitination has only recently begun to be associated with specific cellular processes. However, much remains to be learned about the functional consequences and the translational potential of these non-canonical ubiquitination pathways. There is an obvious need for dedicated tools and methods, including advanced protein mass spectrometry, ubiquitomics, and chemical biology reagents, to expand our knowledge of non-canonical ubiquitination. New workflows will help map reactive sites, identify substrates and functions, and ultimately enable drug discovery and therapeutic development.

This Research Topic aims to expand on the knowledge of research trends in the non-canonical Ub and UBL field by addressing the following themes: 1) new discoveries in non-canonical Ub and UBL events, 2) development and expansion of the ubiquitomics toolbox and methodologies, 3) and of Ub and UBL-oriented chemical biology reagents.

Alonso and Knobeloch contributed an insightful review of the non-catalytic functions of the ubiquitin and UBL proteases. These proteases are essential players in the canonical ubiquitin or UBL conjugation cycles. Consequently, much research has concentrated on unravelling the catalytic activities of these proteases. However, it has been demonstrated that DUBs and UBL proteases possess domains or binding motifs that not only modulate their catalytic prowess but can also mediate entirely distinct functions. Numerous examples, summarised in this review, have shown that either the selective loss of only the protease activity or the complete absence of these proteins can lead to divergent functional and physiological consequences. Furthermore, the authors have emphasized that carefully planned experiments involving the selective inactivation of only the catalytic core or the remarkable rescue experiments with catalytically inactive variants are pivotal in avoiding any potential misinterpretations.

In their contribution, Buzuk and Hellerschmied highlight the critical role of Ub- and UBL-mediated degradation at the Golgi apparatus for protein secretion. Golgi integrity may be compromised in neurodegenerative diseases and certain cancers, resulting in its disintegration and malfunction. Protein ubiquitination serves as an internal protein quality control system to counteract this, facilitating the degradation of aggregated, non-native proteins which may disrupt Golgi integrity. The authors describe two main pathways for this, Golgi Apparatus-Related Degradation (GARD) and Endosome and Golgi-associated degradation (EGAD). The review also describes alternative functions of Golgi ubiquitination, including its role in the regulation of Golgi homeostasis, structural integrity and vesicle trafficking. Finally, the review focuses on the role of Golgi E3 ligases, identifying novel substrates and elucidating the cellular specificity for known Golgi ligases. This increased understanding of the role of Golgi E3 ligases in disease will facilitate the development of molecular PROTACS which can regulate Golgi structure and function.

The review article by van Overbeek et. al. highlights the current state and challenges of studying non-canonical ubiquitination. It emphasizes the gap between *in vitro* studies using recombinant proteins and the *in vivo* functional understanding of these modifications. These *in vitro* methods, often employing lysine-less mutants, are limited and may produce artifacts. For canonical ubiquitination, advanced mass spectrometry techniques like activity proteomics and ubiquitomics are effective for mapping novel substrates and sites, often using peptide-level enrichment with anti-GlyGly antibodies. Non-canonical ubiquitination is even more challenging to study due to its lower abundance and the instability of thioester and oxyester bonds. New tools such as GlyGly antibodies raised against N-terminal sites, the antibody-free StUbEx PLUS method for lysine and N-terminal ubiquitination, and the UbiSite antibody for non-lysine ubiquitinations are discussed. The review calls for further research using chemical biology tools to better understand the biological significance of non-canonical ubiquitination.

Finally, Kloet and van der Heden van Noort’s study introduces an innovative method to investigate *Legionella pneumophila* infection mechanisms by capturing effector enzymes released into host cells. These enzymes, particularly from the SidE family, manipulate host cellular pathways through non-canonical phosphoribosyl serine ubiquitination (PR-ubiquitination), crucial for forming Legionella-containing vacuoles in Legionnaires’ disease. Their approach involves a novel ubiquitin-derived probe with a photosensitive group that binds specifically to catalytic histidine residues in *Legionella* effectors, SidE, and Dup enzymes upon light exposure. The probe, conjugated to a rhodamine-labeled ubiquitin mutant via CuAAC chemistry, was validated through mass spectrometry for efficient activation post-irradiation. Challenges like oxidation and photodegradation were addressed using glutathione (GSH) as a scavenger. Application of this probe successfully crosslinked with *Legionella* effector proteins DupA and SdeAPDE, highlighting its specificity over unrelated enzymes. This chemical biology advancement enhances understanding of PR-ubiquitination mechanisms, potentially informing targeted therapies against *Legionella* infections.

In conclusion, this Research Topic offers a comprehensive overview of recent advancements in the study of non-canonical ubiquitination and UBL modifications. It encompasses detailed insights into the non-catalytic roles of ubiquitin and UBL proteases, emphasizing the ongoing need to deepen our understanding of these alternative pathways, their regulation, and cellular coordination. Additionally, the topic explores the potential of utilising specialised E3 ligases localised within cellular compartments for developing PROTACs to target organelle-specific diseases. Furthermore, the discussion extends to the development and expansion of innovative tools and methodologies, particularly Ub/UBL-oriented chemical probes, which are pivotal for advancing research in this field. By fostering multidisciplinary research, we hope that our Research Topic unravels the complexities and biological significance underlying non-canonical ubiquitination.

